# GAN-based semi-automated augmentation online tool for agricultural pest detection: A case study on whiteflies

**DOI:** 10.3389/fpls.2022.813050

**Published:** 2022-09-16

**Authors:** Christophe Karam, Mariette Awad, Yusuf Abou Jawdah, Nour Ezzeddine, Aya Fardoun

**Affiliations:** ^1^Department of Electrical and Computer Engineering, Maroun Semaan Faculty of Engineering and Architecture, American University of Beirut, Beirut, Lebanon; ^2^Department of Agriculture, Faculty of Agricultural and Food Sciences, American University of Beirut, Beirut, Lebanon

**Keywords:** GAN, data augmentation, pest detection, whiteflies, smart agriculture

## Abstract

Deep neural networks can be used to diagnose and detect plant diseases, helping to avoid the plant health-related crop production losses ranging from 20 to 50% annually. However, the data collection and annotation required to achieve high accuracies can be expensive and sometimes very difficult to obtain in specific use-cases. To this end, this work proposes a synthetic data generation pipeline based on generative adversarial networks (GANs), allowing users to artificially generate images to augment their small datasets through its web interface. The image-generation pipeline is tested on a home-collected dataset of whitefly pests, *Bemisia tabaci*, on different crop types. The data augmentation is shown to improve the performance of lightweight object detection models when the dataset size is increased from 140 to 560 images, seeing a jump in recall at 0.50 IoU from 54.4 to 93.2%, and an increase in the average IoU from 34.6 to 70.9%, without the use of GANs. When GANs are used to increase the number of source object masks and further diversify the dataset, there is an additional 1.4 and 2.6% increase in recall and average IoU, respectively. The authenticity of the generated data is also validated by human reviewers, who reviewed the GANs generated data and scored an average of 56% in distinguishing fake from real insects for low-resolutions sets, and 67% for high-resolution sets.

## 1. Introduction

Agriculture has been a central pillar in the development of humankind in the past and remains a vital driver for local and global economies. Currently, agriculture faces growing pressures with the increasing global population, which is expected to reach over 9 billion by 2050 (Leridon, 2020). With the limited availability of land resources, food security becomes a major issue. On top of that, crop production is severely handicapped by pests and diseases, reducing production by 20–50% annually, with economic losses of up 70 billion US dollars (FAO, 2019). The situation is even more dire in developing countries, where small family farms are responsible for more than 80% of agricultural production (Harvey et al., 2014). These farms often lack the expertise and technology to fight pests and diseases as effectively as industrial farms, and their losses due to these factors can surpass 50% (United Nations Environmental Programme, 2013).

The recent developments in the field of machine learning can help improve the diagnosis of plant pests and diseases through traditional visual assessment methods. Convolutional Neural Networks (CNN) have achieved superior performance in image classification and object detection tasks (Liu et al., 2022). Hughes and Salathe (2015) used the PlantVillage dataset to train a GoogLeNet convolutional network achieving a 99.34% accuracy by using transfer learning from a model pre-trained on the ImageNet dataset (Mohanty et al., 2016). Despite the high accuracy, there are no further experiments to prove the effectiveness of such classification models out in the field, due to the plant leaf images being taken in a controlled lab setting. Türkoğlu and Hanbay (2019) achieve an accuracy of 97.86% using a ResNet50 CNN and an SVM classifier, compared to only 70.90% for their best shallow-feature model, justifying the need for deep feature extractors.

In more difficult object detection tasks, Gutierrez et al. (2019) benchmark different feature extractors and detector architectures using scouting robots, to detect two tomato whitefly species in their egg and adult insect stages (four classes in total), in 54,743 images. Their RCNN model can detect the two insect stages with a 53% accuracy, but performs poorly on egg stages, with an accuracy of around 8%. Another similar study (Fuentes et al., 2017) benchmarks different architectures and feature extractors on a dataset of tomato plants, examining nine different classes of pests and diseases. Their dataset contains 5,000 images and more than 43,000 labeled objects. They compare the performance of Faster R-CNN with different feature extractors (VGG and ResNet) to that of SSD-ResNet and R-FCN-ResNet. The R-FCN-ResNet50 model achieves the highest accuracy with 86% mAP, and notably, an AP of 0.94 on the whitefly class, albeit on only 49 images with 404 insects. Selvaraj et al. (2019) use similar architectures to perform disease detection on banana crops, creating robust models with 18 disease classes in 18,000 field-captured images.

Object detection tasks are more difficult to solve, require higher computation loads, and need fine-grained annotations which are more expensive to obtain on specialized datasets. While computing power at the edge increases, the ever-present need for large amounts of data continues to be an obstacle to the development of high-performance models. Deep neural networks need a lot of data to achieve accuracy and robustness, but this comes at the cost of more computing resources spent on training (Rizk et al., 2019), and more human resources spent on data collection and labeling. Generative Adversarial Networks (GANs) have been used to help mitigate this problem of data hunger in recent years through synthetic data generation, but they come with their own sets of challenges such as the computing power needed to generate high-resolution images and the difficulty of generating fine-features at low-resolution. The medical field has made use of these architectures to overcome the data scarcity problem: Motamed et al. (2021) leverage GANs to generate synthetic data, boosting the performance of pneumonia and COVID-19 in X-rays, especially when compared to traditional augmentation techniques, such as zooming and rotating. These methods are also leveraged in the agricultural field, where Bi and Hu (2020) use a Wasserstein generative adversarial network with gradient penalty to avoid overfitting on a limited dataset, and it is shown to improve plant disease classification by around 24%.

To this end, this work proposes the use of a novel, GAN-based pseudo-automated pipeline for data augmentation, thereby leveraging synthetic data generation in order to increase dataset sizes, decrease data collection, and improve the performance of lightweight CNNs for detecting and counting large numbers of small pests on plant leaves.

This project combines the two tasks of (1) progressively building a dataset and (2) building a learning model for multiple objects types which are very small and very numerous in each image. This is what mainly sets this work apart from others. In Ramcharan et al. (2019), and most other works that target disease detection, the objects are disease symptoms which are large and less numerous in each image, making the task relatively easy. Researchers in Gutierrez et al. (2019) obtain good results on the disease-objects in their dataset, and on the single pest-object, whiteflies. However, this performance might be unreliable due to the overfitting on the small dataset of 49 images and 404 whiteflies. Our work separates itself by taking on the challenging task of detecting small objects in large numbers, and overcomes the overfitting and under-performance problems faced by limited datasets through synthetic data generation, and the hybrid use of GANs in combination with human labeling and expertise to produce authentic images. The work also uses data collected out in the field instead of a controlled lab setting, which should make the models more robust and more-readily deployable in real-world applications. Our work culminated in building an open-access tool that researchers in the field can adopt for developing their machine learning models for pest detection using a minimal set of real images.

The proposed novel augmentation pipeline is shown to improve the recall of a YOLO-based object detector for a pest-counting task by more than 38% points, by increasing a small dataset size four-fold. The data generation interface is flexible and applicable to a wide array of object detection tasks, especially improving performance when dealing with small dataset sizes. It allows users to generate synthetic data from existing images and objects, and is validated by a thorough human assessment of the authenticity of the generated data.

**Figure 1:**
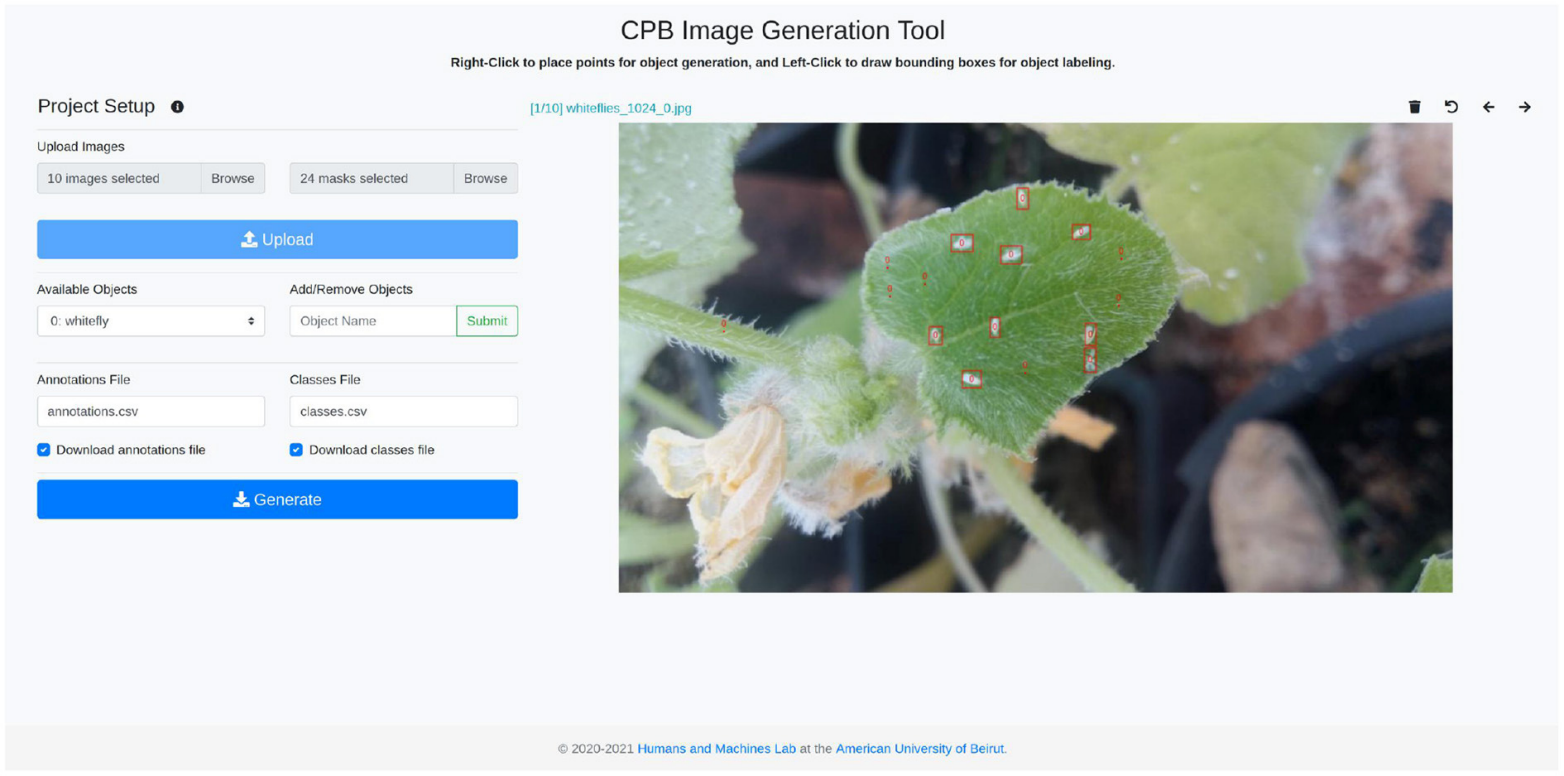
Copy-paste-blend tool for synthetic data generation.

This novel image generation tool, which is publicly available at: https://github.com/ChristopheKar/cpb-gen, can then be used to augment small datasets, which can then be used to train accurate yet lightweight object detection models. Its annotation and generation interface is shown in Figure 1. The datasets and models can be developed, trained, and deployed by low-resource individuals on low-resource devices for a myriad of object detection tasks, and more specifically, to detect pests on plant leaves. Early and accurate detection of plant pests will allow both small and large-scale farmers to timely treat the plants, improving crop yield and production.

## 2. Methodology

This section will breakdown the different parts of the augmentation pipeline, from the datasets used to the different networks for image generation and object detection.

### 2.1. Dataset

The dataset is collected from the American University of Beirut's greenhouses, from multiple locations. The images are taken in the field so that the background and lighting variations correspond to real-world conditions, contributing to the robustness of the application. The images are captured using cell-phone cameras at different scales. The dataset contains the whitefly species *Bemicia tabaci* on five different crops: tomato, eggplant, pepper, beans, and cucumber. The dataset contains a total of 770 images with around 3,400 labeled whitefly objects. A training set of size *D*_base-train_ = 560 is chosen as the base dataset for the work, containing 2,520 whiteflies, along with a validation set *D*_base-valid_ = 70 and a test set *D*_base-test_ = 140. The validation and testing sets are fixed throughout all experiments, whereas the training set size varies to showcase the effect of data generation and augmentation.

The main task is counting the number of adult *B. tabaci* whiteflies on plant leaves. The *B. tabaci* whitefly is considered the second most widespread and economically-damaging arthropod pests, attacking an estimated range of 36 plant genera throughout 156 countries (Willis, 2017), notably affecting tomato and cotton crops, but also beans, cucurbits, peppers, cassavas, and okra (Goolsby et al., 2005). *B. tabaci* is distributed worldwide and has been rapidly spreading during the past 15 years, but is especially damaging in the tropical and subtropical regions. In addition to its feeding damage, *B. tabaci* is also a vector of more than 100 plant viruses, of which Begomoviruses cause the most damage, leading to crop yield losses ranging from 20 to 100% (Jones, 2003).

### 2.2. Data augmentation

Due to the extensive work that is necessary to maintain a colony of pests of different species on different crop types, collect enough images, and label each small pest with a bounding box, data augmentation is a crucial step in the pipeline toward achieving accurate detection of pests. Due to the difficulty of generating high-resolution images with fine-grained features or small objects using GANs, such as insects in our case, we have resorted to a semi-automated technique of synthetic data generation, supported by GANs and operated by humans. Noting the lack of existing augmentation tools, we have developed an image labeling and generation interface using Python, OpenCV, and Flask, accessible through a webapp, which will allow users to produce realistic pest-infested leaf images starting with a small dataset.

*Note: In the abbreviated terms below, the first subscript refers to the image data:* ***o****bjects*, ***m****asks, or whole* ***i****mages. The second subscript refers to the items'* ***i****nitial or* ***f****inal state*.

The augmentation workflow is as follows:

- Create segmentation masks for objects (pests) of interest, preferably from multiple examples for each class (species), with *N*_*mi*_ as the number of initial or source object (pest) masks.
- Optionally augment these object masks using GANs to increase the final source pool size to *N*_*mf*_ GAN-augmented object (pest) masks.
- Prepare a small dataset of background (leaf) images, with or without existing objects (pests), acting as a base/source dataset to augment, with *N*_*ii*_ initial images and *N*_*oi*_ initial objects.
- Label existing objects (pests) by drawing bounding boxes.
- Add new objects to the images by choosing one or multiple locations for each object type to be generated.

Following this workflow for our use-case, the tool will produce a final augmented dataset of *N*_*if*_ leaf images with a total of *N*_*of*_ artificially pasted pests, along with files that store the annotations and class names. The tool thus allows users to generate and label synthetic data from existing images and objects to train and develop detection models.

**Figure 2:**
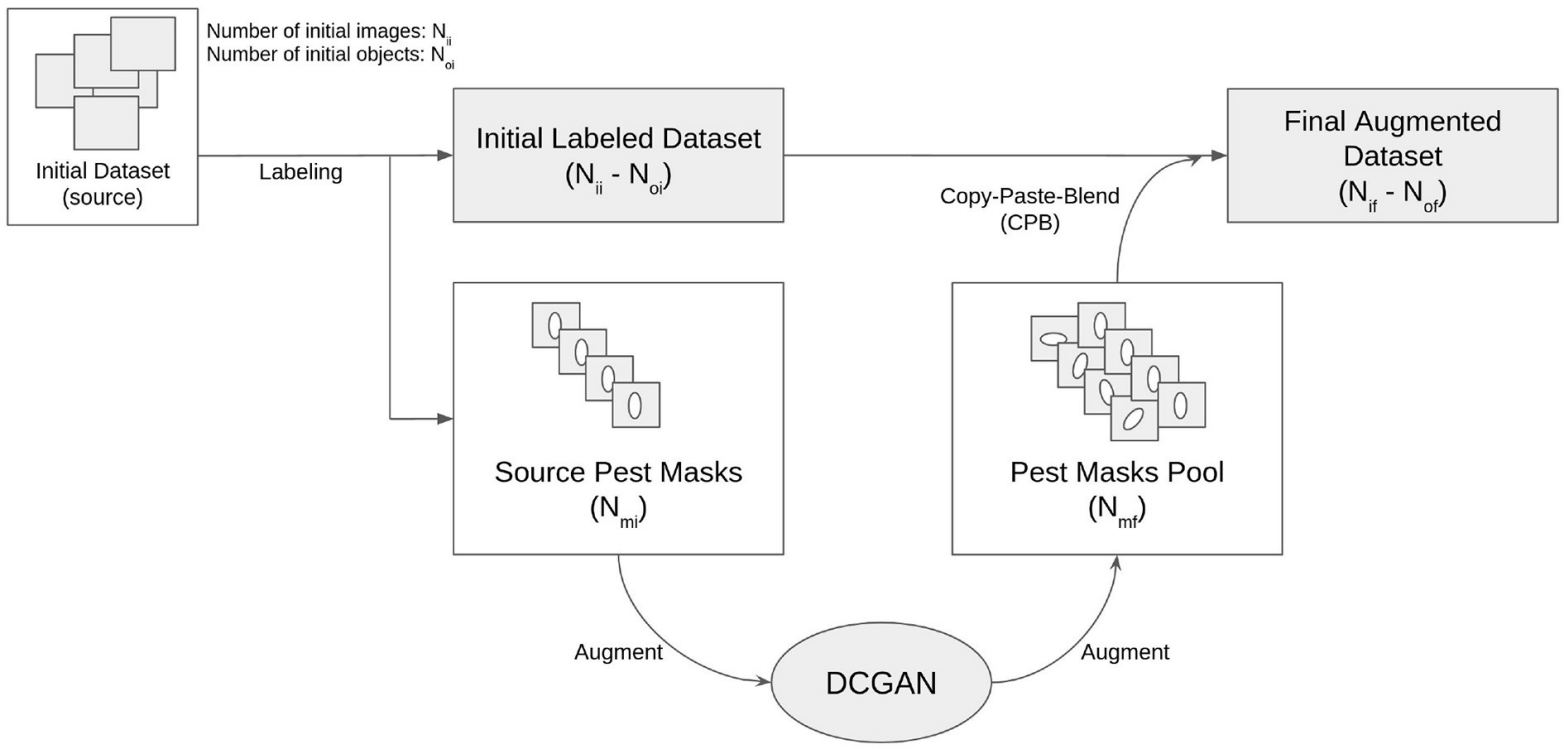
Copy-paste-blend + DCGAN augmentation pipeline (CPB+GAN).

**Figure 3:**
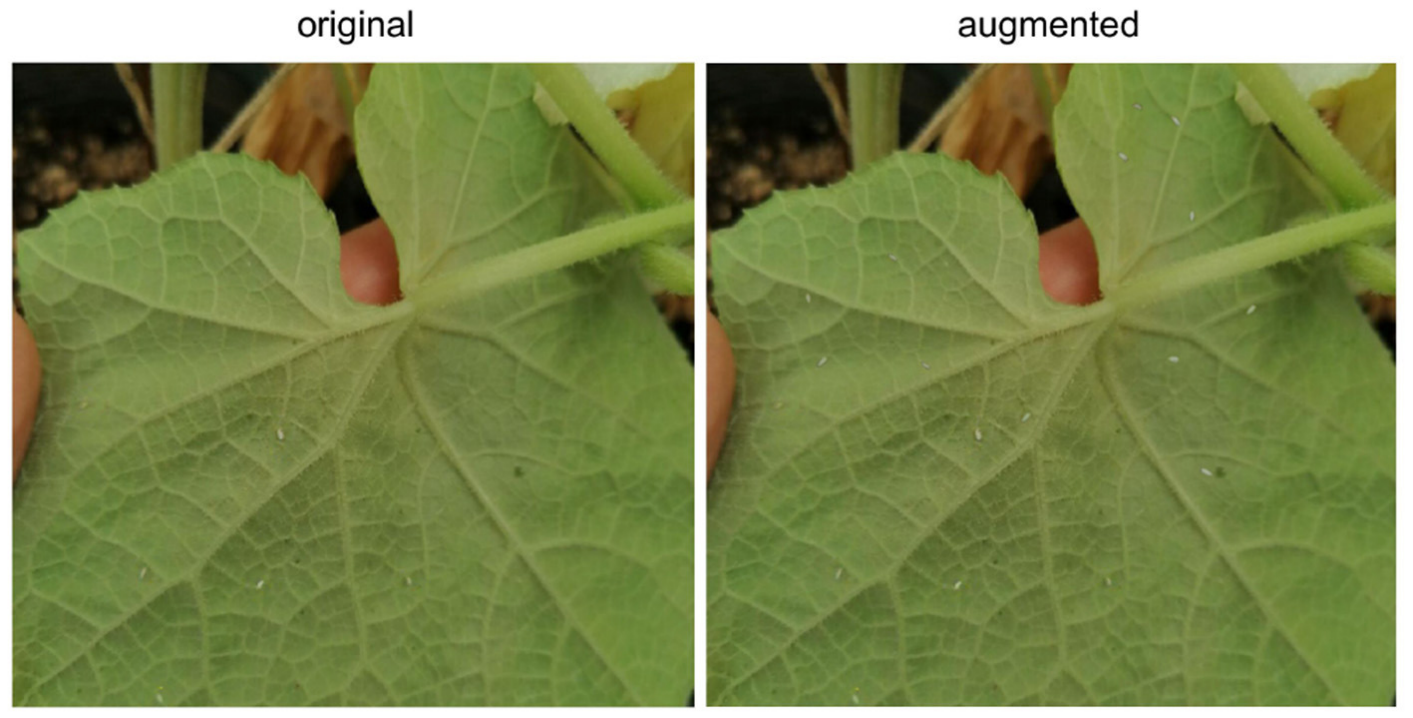
CPB+GAN output comparison.

The result is a pipeline for generating artificial images of pest-infested leaves, as shown in Figure 2. The augmentation stage can be simplified and summarized by a three-step process: copying the source insect based on its mask, pasting it on the destination leaf image in a location chosen by a human reviewer, and blending the source insect with its destination background to maximize realism. This process will be referred to as Copy-Paste-Blend (CPB). In order to further diversify the augmented results, we introduce an additional step at the beginning of the pipeline: the initial size of the source mask pool is increased by generating new objects, thereby generating completely new insect masks not previously seen in the base dataset and diversifying the end-images. A Deep Convolutional Generative Adversarial Network (DCGAN) (Radford et al., 2015) is thus used to generate images of size 32x32 representing individual pests of varying sizes. The entire pipeline will then be referred to as **CPB+GAN** if it includes this initial augmentation stage, and **CPB-NoGAN** if it does not generate new insect masks. A sample augmentation is shown in Figure 3.

#### 2.2.1. Generative adversarial network

The DCGAN used for augmenting the pest masks has a generator made up of three up-sampling blocks each with an up-sampling layer, a convolution layer with ReLu activation, and a batch normalization layer, and a final convolution layer with a hyperbolic tangent activation, outputting a 32 × 32 RGB image of a whitefly. The discriminator has three convolutional blocks using ReLu activation and dropout, as well as batch normalization on the last two blocks. The final layer is a fully-connected sigmoid activated layer. The entire network is optimized using Adam with a learning rate of 0.0002.

### 2.3. Object detection

For detecting pests on the leaf images, the single-stage, real-time YOLO networks are used for the experiment iterations. We consider the YOLOv3 (Redmon and Farhadi, 2018) model as well as its lightweight version, YOLOv3-tiny, in addition to a custom YOLO-based PestNet. YOLOv3 is a performant object detection model that can produce high-accuracy results while running at a high frame-rate, providing detections in real-time (>24 frames-per-second). Real-time detection is important because it allows users to receive instantaneous results in the field, and because it means that the network will still be usable even when ported to low-resource devices. The modified YOLO network, dubbed PestNet, is based off the DeepSperm network by Hidayatullah et al. (2020), whose task was to achieve a real-time bull-sperm cell detection in densely populated microscopic observation videos. The original DeepSperm network uses 29 convolutional layers, a dropout layer, and a final detection layer combined with image augmentation to prevent overfitting. It was modified through the addition six convolutional layers and a weak dropout layer with *p* = 0.2, and called PestNet. Whitefly detection and bull sperm cell detection are similar tasks due to the small size of the objects and the large numbers in which they are present in images.

### 2.4. Tool validation

To evaluate the realism of the generated images and the tool's effectiveness, reviewers from two different backgrounds, agricultural or image processing, were asked to visually assess real and synthetic datasets and label each image as real or artificial, but also to count the number of fake occurrences (generated pests). They provided reasons as to why certain objects were deemed unauthentic, in order to understand the shortcomings of the pipeline and the areas that need improvement.

## 3. Experimental results

### 3.1. Experimental setup

All experiments were performed on a Tesla V100 GPU with 32 GB of VRAM, using a batch size of 64 subdivided into four mini-batches of size 16, using a resolution of 512 × 512 pixels for all images. The models were trained for 3,000 batches, equivalent to about 81 full epochs. All object detection networks were trained using the Darknet framework (Redmon, 2013–2016), while the generative networks were trained using Keras and Tensorflow (version 1) (Abadi et al., 2015).

### 3.2. Baseline performance

The first batch of experiments aims at evaluating object detection models on the raw non-augmented dataset to provide a baseline performance which can be referenced when comparing the different augmentation techniques and experiments.

The baseline models are all validated and tested on the same sets, *D*_base-valid_ = 70 images and *D*_base-test_ = 140 images, as stated in Section 2.1. The training is done on two subsets of the base dataset, one of size 560 images (*D*_base-train-560_) and another of size 140 images (*D*_base-train-140_).

These baseline results are shown in Table 1. Counting the number of insects on the leaves represents the essence of this pest detection task, and as a result, recall constitutes one of the most important metrics to evaluate the performance of the models. Drawing extremely accurate bounding boxes around the pests is not as essential as detecting all the pests present on the leaf. The recall metric is thus used at two different Intersection-over-Union (IoU) thresholds, at 0.50 and 0.75 (R@.50 and R@.75). In other words, the IoU threshold defines if a detection is counted as correct (positive): if the ratio of the areas of overlap between the detected bounding box and the groundtruth bounding box is higher than the threshold fraction, the detection is counted as true. The Average Precision (AP) metric also thresholds by IoU, but combines information from both recall and precision for a more comprehensive evaluation. Finally, the model size and average training time per batch of images allow us to get an idea of the resource-consumption of the model. The YOLOv3 model exhibited the best performance when trained on *D*_base-train-560_, followed closely by PestNet. YOLOv3 only outperforms PestNet by 1.3, 7.3, and 1.9% in R@.50, R@.75, and average IoU, respectively. On the other hand, the PestNet model size is more than 17 times smaller than YOLOv3, which will make it much easier to load on low-end devices. YOLOv3-Tiny performs badly across the board despite being comparably light to PestNet. When trained on *D*_base-train-140_, PestNet seems to generalize better than YOLOv3 and outperforms it by a slight margin, scoring 2.6, 11.7, and 4.8% higher than it in R@.50, R@.75, and average IoU, respectively, despite being a smaller model. The performance of both models dropped by almost 50% on all metrics when the training set size dropped from 560 to 140 images.

**Table 1:** Baseline YOLO performance (no augmentation).

**Network**	**Training size**	**R@.50**	**R@.75**	**Avg IoU**	**Model size (MB)**	**Train. time/Batch (s)**
YOLOv3	560	**0.955**	**0.603**	**0.752**	245	6.68
YOLOv3-Tiny	560	0.528	0.135	0.472	31	**5.00**
PestNet	560	0.943	0.562	0.738	**14**	5.27
YOLOv3	140	0.532	0.214	0.330	245	6.65
YOLOv3-Tiny	140	0.196	0.042	0.101	31	**4.98**
PestNet	140	**0.544**	**0.239**	**0.346**	**14**	5.31

Best metrics are shown in bold.

### 3.3. Data augmentation

To evaluate our data generation pipeline, the different augmentation variations are tested on the PestNet model because of its superior balance between accuracy and speed. PestNet is able to offer accuracy on-par with that of YOLOv3 while being a much smaller and lighter model.

The first variation is the CPB-NoGAN version of the pipeline, augmenting the starting dataset size with the original set of pest masks of size *D*_*ps*_ = 20. The second variation is coupling the CPB pipeline with an initial augmentation of source pest masks using the DCGAN (CPB+GAN) to yield a usable mask pool of size *D*_*pf*_ = 60.

**Figure 4:**
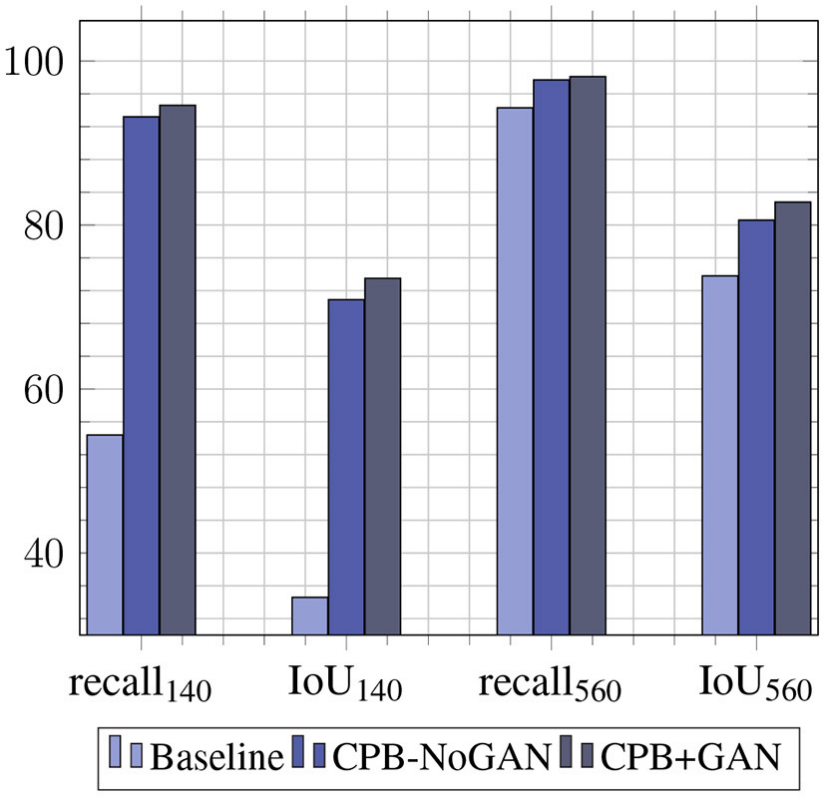
Performance gains of PestNet with CPB for Recall@.50 and Average IoU.

The augmentation pipeline is applied on the two sets used for the baseline training: (1) *D*_base-train-140_ is augmented four-fold for a final size of 560 images. (2) *D*_base-train-560_ is augmented two-fold for a final size of 1,120 images. Each of these sets is thus augmented twice, once with the original object mask pool of 20 images (CPB-NoGAN), and once with the GAN-augmented object mask pool of 60 images (CPB-NoGAN). Note that all datasets are multiples of a fundamental or base dataset size of *D*_*b*_ = 140 images. These iterations are summed up in Table 2, along with the performance results. It is interesting to examine the performance gains to each metric brought forth by each part of the pipeline: the simple increase in the number of images (CPB), and the increase in the diversity of the inserted objects (CPB+GAN). The Recall@.50 will mainly measure the number of detected pests (sensitivity), while the average IoU will mainly indicate the precision with which the bounding boxes are drawn. This comparison is shown in Figure 4 shows that augmenting the dataset size using CPB-NoGAN from 140 to 560 images brings PestNet's recall metric close to the baseline model trained on 560 images (within 1.2%), while the average IoU does not quite reach the same level as before (still 3.9% away). This may be due to the limited mask pool size, which results in the same 20 whiteflies being added to all the images. This restricted diversity in the augmented images leads to reduced performance when compared to a model trained on an original dataset equal in size to the augmented dataset: the original dataset will be more varied in terms of objects (insects) it contains. This problem is solved through the use of GANs to augment the starting mask pool from 20 to 60 masks. The increase in diversity in the final augmented dataset will lead to better generalization on the test set, contributing to the precision of the detected bounding box. The graph in Figure 4 clearly shows how the Average IoU metric is more sensitive to the dataset's diversity than the recall metric: the performance gain in average IoU from CPB-NoGAN to CPB+GAN is at 2.2% points, while the increase in recall between the two pipelines is only at +0.4% points, for the original dataset size of 560 images.

**Table 2:** PestNet test performances for different CPB configurations.

**Pipeline**	**Original dataset size**	**Augmented dataset size**	**Pest masks pool**	**Recall @.50**	**Average IoU**
CPB-NoGAN	140 (*D*_s_*1)	560 (*D*_s_*4)	20	0.932	0.709
CPB+GAN	140 (*D*_s_*1)	560 (*D*_s_*4)	60	0.946	0.735
CPB-NoGAN	560 (*D*_s_*4)	1120 (*D*_s_*8)	20	0.977	0.806
CPB+GAN	560 (*D*_s_*4)	1120 (*D*_s_*8)	60	0.981	0.828

### 3.4. Human visual assessment

The image generation pipeline is not only validated by the performance boost of the detection model, but also by a visual assessment of the authenticity of the generated images by humans. The reviewers are 19 students (11 females and 8 males) in the same age group of 20–25, but come from two different backgrounds: computer science/vision (13) and agriculture (6). The technical background comes into play when evaluating artificial plant and pest images as the reviewers from agricultural backgrounds are better trained and equipped to recognize anomalies in the synthetic data. The reviewers assessed a total of three artificially-generated sets of 60 images each. The first two sets were generated using the CPB-NoGAN pipeline, from 20 original whitefly objects, at a lower-resolution of 512 × 512 pixels, and a higher-resolution of 1,024 × 1,024 pixels. The third set was generated using the CPB+GAN pipeline, from 20 original whitefly objects augmented to 60, at the resolution of 512 × 512 pixels for the images. The 512 × 512 resolution corresponds to the models' resolution for a fair comparison, but at this size the anomalies in the synthetic data are more difficult to detect for humans.

**Figure 5:**
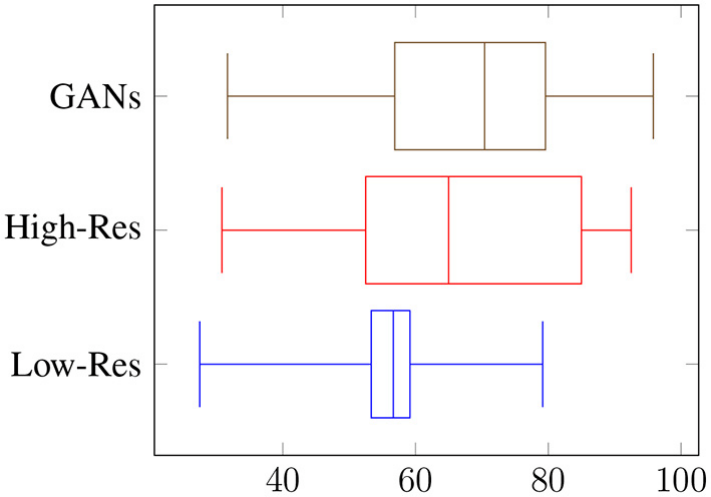
Visual assessment of generated datasets: real/fake classification accuracy (%).

**Figure 6:**
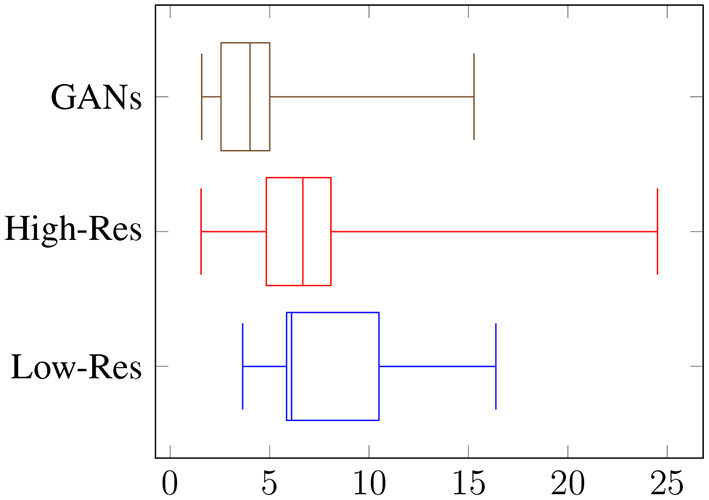
Visual assessment of generated datasets: RMSE on number of fake occurrences.

Overall, the reviewers were able to distinguish fake objects with an average accuracy of 56% at low resolution, and 67% at high resolution, as shown in Figure 5. When asked to count the numbers of fake occurrences in an image, the accuracy was 26% with an RMSE of 8.2 for low resolution, and an accuracy of 42% with an RMSE of 7.3 for high resolution, as shown in Figure 6. Clearly, the task is easier at higher resolution, but the authenticity of the generated data can be validated by low overall performance of the reviewers on these two sets. It is interesting to note the difference in performance between the vision-background students and the agriculture-background students: the top reviewer is a researcher in agriculture and can distinguish real from fake images with an accuracy of 79 and 91% for low and high resolution, respectively, and can count the number of fake occurrences with an accuracy of 42 and 72%. Compared to the average reviewer, This is an increase of around x1.4 for binary classification of real/fake, and x1.6 for counting.

### 3.5. Discussion

We have shown results for baseline YOLO performances, and the effect of different data augmentation pipelines on these performances. The baseline YOLO performances justify the use of PestNet, since it is a lightweight object detection model, capable of almost matching YOLOv3's performances while still being a much smaller model, which makes it more likely to be implemented in applications on low-cost devices. The merits of PestNet are further justified by the failure of YOLOv3-Tiny, another lightweight model, to produce similar results. The main focus of this work is improving these baseline performances, through the use of the data augmentation pipeline, dubbed CPB. This pipeline allows us to increase the dataset sizes by reproducing the objects of interest (whitefly pests) on different plant leaves, making the model more robust by diversifying the examples it sees. As a result, augmenting the training set from 140 to 560 images using CPB-NoGAN yields a jump by 38.8% points for R@.50, almost matching the performances of a model trained on 560 original images. This highlights the need for such pipelines in scenarios where the data is limited. While CPB-NoGAN boosts both the recall and average IoU metrics, the CPB+GAN pipeline allows us to generate new pests, increasing the diversity of the objects of interest, and further boosting the resulting average IoU, which measures the precision in the bounding box detection. Therefore, starting from 140 images and applying the CPB+GAN augmentation, we can match the performance of a model trained of 560 images from the start. The augmentation pipeline has therefore fulfilled its function in decreasing the effect of data scarcity. Finally, the human reviewers serve to assess and help explain both the benefits and shortcomings of this pipeline. At the model's resolution, the reviewers can distinguish real from fake images with an accuracy of 56%, which means that the generated are realistic enough. At higher resolutions, this accuracy goes up to 67%, which means the images are still somewhat realistic, but are now easier to spot and may not be as effective in portraying real-world scenarios. The augmentation pipeline is then well-suited for training at a resolution 512 × 512, but should probably be improved before being used with higher-resolution models. However, it is important to note that most datasets do not include images at resolutions of 1,024 × 1,024, and that using lower resolutions can help with model portability and computational loads on low-end devices. The reviewers pointed out that potential areas of improvements include refining the blending process to reduce pest outline and color issues. Overall, these results justify the use of the PestNet model for this pest detection task, as well as the CPB+GAN pipeline for generating synthetic datasets. Compared to the literature, this work differs from the literature in its use of non-traditional augmentations, leveraging GANs and a semi-automated process to artificially generate new objects of interests in new configurations on different backgrounds. It also differs in its use of lightweight architectures rather than larger and slower models like the popular ResNet + Faster-RCNN for object detection, keeping portability and embeddability. Finally, it also stands out in the difficulty of the task at hand: detecting small numerous pests on a plant leaf is harder than detecting diseases whose symptoms take up a large portion of the leaves, or larger pests that are more prominent in the images, and present in fewer numbers. One shortcoming, which can also be addressed to other works in the literature, is the difficulty of comparing the detection performances of different models, due to the inherent differences in the studied crops, diseases, and pests, and the unavailability of public datasets that target similar object detection tasks.

## 4. Conclusion

In this work, we developed a synthetic data generation pipeline and online tool leveraging GANs, that can be used to augment small datasets in order to achieve accurate object detection with lightweight models. The publicly accessible tool allows even inexperienced users to create large datasets starting from a few real images. Developing simple tools contributes to enhanced food security by allowing all stakeholders in agriculture, whether big or small-time farmers, to optimize their pest management strategies and reduce the yield loss incurred by pest attacks.

The validity of the pipeline and accompanying tool was tested on an pest detection task for different sizes of a *B. tabaci* whiteflies dataset, collected at the American University of Beirut's greenhouses. Data generation allowed us to augment a dataset of 140–560 images, increasing the performance of a lightweight object detection model, PestNet, by 38.8% points in R@.50, and by 36.3% points in average IoU. When GANs are incorporated as a method to augment the starting object masks (pests) inserted into images, the recall and average IoU are further increased by 1.4 and 2.6% points, respectively. The GAN variation of the pipeline especially aids in improving the precision of the detected bounding box by boosting the generalization of the model on the test set due to the increased variety in the introduced augmentations. Thus, starting from only 140 images and using data augmentation, we were able to match the performance of a model trained on 560 images.

The tool's validity is also tested through visual assessment of its image outputs, conducted by 19 student reviewers from agricultural or machine-learning backgrounds. This review serves to evaluate the authenticity of the augmented images and to identify its weaknesses for further improvement. When presented with a dataset with 512 × 512 RGB images, reviewers were only able to classify the images are real or generated with an average binary accuracy of 56%, with the best reviewer achieving 79% accuracy (agricultural background). On the more challenging task of counting the number of fake objects in each image, the binary accuracy was only 26%, with an average RMSE of 8.2. The pipeline is then well-suited for realistic data generation, but could still be improved.

The lightweight model and the small dataset size we use are both indications that this work may be beneficial for real-world applications on low-end devices. However, some shortcomings of this study include the difficulty of comparison with the literature for similar tasks due to the unavailability of data, and focus on the detection of only one pest type, the whitefly. Future work could then target the expansion of the existing dataset to include more pests, to strengthen model robustness, range of applications, and validation strength. The CPB pipeline could be improved as well by refining the blending process to reduce pest outline and color issues, marked as being the top indicators of a fake pest by reviewers. Finally, additional work could target the augmentation pipeline and tool to include additional features such as model training and deployment, further bridging the gap between model development and the end-users, which are small-time farmers interested in improving their crop health with early pest detection.

## Data availability statement

The raw data supporting the conclusions of this article will be made available by the authors, without undue reservation.

## Author contributions

CK developed the software, ran the experiments, and produced a draft of the manuscript. MA directed the research, identified the framework to be implemented and helped in the experimental setup, and revised the manuscript multiple times. YA co-directed the research, provided input on the pest cycle, and corrected final versions of the manuscript. NE collected the agriculture data, labeled it, and helped in the human annotation as well as revising the manuscript. AF collected the agriculture data, labeled it, and contributed to the manuscript write-up. All authors contributed to the article and approved the submitted version.

## Funding

This research was supported by WEFRAH whose focus is collaboration to achieve primary resources security. WEFRAH aims to make the American University of Beirut a regional hub for innovations in water-energy-food-health nexus and interconnectedness in renewable resources in arid and semiarid regions.

## Conflict of interest

The authors declare that the research was conducted in the absence of any commercial or financial relationships that could be construed as a potential conflict of interest.

## Publisher's note

All claims expressed in this article are solely those of the authors and do not necessarily represent those of their affiliated organizations, or those of the publisher, the editors and the reviewers. Any product that may be evaluated in this article, or claim that may be made by its manufacturer, is not guaranteed or endorsed by the publisher.
